# Health worker delivered Contact and Safety Planning (CASP) for suicide prevention in Chhattisgarh, India: Protocol for a non-randomized, controlled pilot study

**DOI:** 10.12688/f1000research.159494.2

**Published:** 2025-10-06

**Authors:** Sonali Kumar, Sapna Negi, Snehasish Tripathy, Derek deBeurs, Deepa Pandit, Dr Ben Wijnen, Dr Lakshmi Vijayakumar, Laura Shields Zeeman, Soumitra Pathare

**Affiliations:** 1 Centre for Mental Health Law & Policy, Indian Law Society, Pune, Maharashtra, 400005, India; 2 University of Amsterdam, Amsterdam, The Netherlands; 3 Trimbos-instituut, Utrecht, The Netherlands; 4 SNEHA, Chennai, India

**Keywords:** Suicide prevention, India, Safety planning, Brief interventions, Health system

## Abstract

**Background:**

India records the highest number of suicide deaths globally, but suicide prevention efforts are hindered by a lack of trained personnel within the public health system. Given that an index suicide attempt is a strong predictor of future suicide, intervening with individuals who have recently attempted suicide is a targeted prevention approach that can be delivered within the public health system and has potential to be scaled up across low-resource settings.

**Aim:**

To test the implementation and preliminary effectiveness of Contact and Safety Planning (CASP) in reducing suicidal behaviour and symptoms of depression among adults with a recent suicide attempt in Chhattisgarh.

**Methods:**

We will carry out a non-randomized, controlled pilot study to evaluate the feasibility, acceptability, adoption, reach, implementation (including cost) and preliminary effectiveness of CASP when delivered by health workers – emergency nurses and Community Health Officers – in two districts of Chhattisgarh (n=250). The control group will receive Enhanced Usual Care, consisting of telephonic counselling by trained District Mental Health (n=250) program staff. Data will be collected at baseline and at 6- and 12-month follow- up.

**Conclusion:**

This study will shed light on the feasibility of CASP and inform its further refinement to address suicide at scale in India.

**Trial Registration:**

Clinical Trials Registry India (CTRI/2022/12/048087) dated 1 October 2022. Link here.

**List of abbreviations**
BIC: Brief Intervention and Contact
CASP: Contact and Safety Planning
CHC: Community Health Center
CHO: Community Health Officer
CSSRS: Columbia Suicide Severity Rating Scale
DMHP: District Mental Health Plan
EUC: Enhanced Usual Care
EQ-5D-5L: EuroQol-5D-5L
HWC: Health and Wellness Center
NCRB: National Crime Records Bureau
PHC: Primary Health Centre
PHQ-9: Patient Health Questionnaire-9
RMA: Rural Medical Assistant
SC: Scheduled Caste
TiC-P
: Treatment Inventory of Costs in Patients
WHO: World Health Organisation

## Introduction

Suicide is a leading threat to the well-being of communities globally, and particularly in India which reports the highest number of suicide deaths in the world. ^1^ Although Indians make up 18% of the world’s population, India accounts for 26.6% of suicides globally. ^2^ Since 2017, suicide rates in India have increased steadily with suicide ranking as the first and second leading cause of death among young women and men aged 18-39 respectively. ^3, 4^ Data from the National Crime Records Bureau (NCRB) indicates an upward trajectory, with a national suicide rate of 12.4 per 100,000 individuals in 2022 up from 12 in 2021, marking the highest rate in NCRB history. ^4^ This is despite a recognized under-reporting of suicides in India by as much as 30-100%. ^3, 5^

Chhattisgarh, a central Indian state, has a suicide rate of 28.2 per 100,000 (2022), the fourth highest among states in India and more than double the national suicide rate of 12.4 per 100,000. ^4^ The Chhattisgarh District Mental Health Program (DMHP) was designed to offer telephonic and in-person follow-up counselling for individuals who have attempted suicide; however, most DMHP clinics are unable to implement the intended program due to a dearth of specialized providers, such as psychiatrists, psychologists or counsellors trained to respond to suicide. Consequently, in many districts, there is no follow-up by a professional after a suicide attempt, despite prior research demonstrating that a previous suicide attempt is one of the strongest predictors of future attempts. ^6, 7^

Although the State Government of Chhattisgarh is drafting a State Suicide Prevention Strategy (not yet published); the lack of human resources is a significant constraint in the state which necessitates the mobilization of public health resources to implement suicide response and prevention measures. Contact and Safety Planning (CASP) is an adaptation of brief intervention and contact (BIC) and safety planning interventions, methods which have been effective in reducing suicidal behavior among high-risk individuals worldwide. ^7– 10^ CASP was developed and adapted from evidence-based safety interventions for the Indian context by SNEHA, Chennai and showed to be effective, feasible and acceptable when delivered among a refugee population in Tamil Nadu. ^8^ BIC has been effective in reducing repeat suicides in a 5-country study ^11, 12^ and is recommended by the World Health Organisation (WHO) as an effective intervention to reduce suicides and attempted suicides in the general population. ^13^ Safety planning interventions have been found to reduce fatal and non-fatal suicide attempts in a recent meta- analysis ^9^ and when tested in conjunction with brief contact among veterans in the US. ^14^ Leveraging the public health system to deliver CASP may be a promising solution to address the treatment gap for suicide in Chhattisgarh and similarly low-resourced settings, however, the feasibility and acceptability of a health-systems approach for CASP is yet to be determined.

This study will integrate CASP into the public health system by training non-specialist health workers to identify and follow up with adults who have recently attempted suicide in two districts where the DMHP is not providing any response to suicide attempts (due to personnel shortages). We will train two groups of health workers: (1) nurses in emergency wards of health centres to identify and recruit adults who have attempted suicide from emergency wards, and (2) Community Health Officers (CHOs) at community-based Health and Wellness Centers (HWCs) to deliver the CASP intervention in the community over an 11-week follow-up period.

Follow ups by CHOs will include suicide risk assessments, provision of emotional support and empathetic listening, and collaborative safety planning to identify steps a participant can follow if they are in distress. Steps include identifying symptoms of distress, recognizing coping mechanisms and planning for future use of coping strategies, activating support networks, referrals for professional care and sharing details of the nearest emergency services. The first follow up visit will occur within one week of discharge from a healthcare facility post-attempt, and then continue periodically over the immediate 11-week period afterward.

## Protocol

### Aims

The primary aims of this study are to assess the feasibility, acceptability, reach, adoption and implementation (including cost) of delivering the CASP intervention within the public health system in Chhattisgarh.

The secondary aim is to assess:

• Difference in rate of suicide re-attempt at 6 and 12 month follow up between intervention and control groups,
• Preliminary impact on suicidal behaviour and symptoms of depression at 6 and 12 month follow up within the intervention group only.

For the purposes of this study, suicidal behaviour is defined as inclusive of suicidal intention, ideation, planning and fatal or non-fatal suicide attempt.

### Study design

The CASP intervention will be tested in the form of a 1:1 non-randomized, controlled pilot study. The pilot study will consist of two arms, comparing the CASP intervention in the two districts of Balod and Rajnandgaon against Enhanced Usual Care in the two districts of Mungeli and Balodabazar.

### Setting

The Central Indian state of Chhattisgarh, formerly a part of Madhya Pradesh, was granted statehood in 2000 and is the ninth largest state in India (estimated population 25.5 million). ^15^^,^^16^ 75% of the population live in rural areas and the state has the highest tribal population among large states in India with one-third of its population belonging to tribal communities. ^16, 17^ Chhattisgarh is divided into 32 districts as of 2021, 28 of which are covered by the Government’s District Mental Health Program (DMHP). ^18^ In 2022, Chhattisgarh contributed 4.9% of India’s suicide burden, with a rate of 28.2 per 100,000 people, more than twice the national average (12.0). ^4^ According to the National Mental Health Survey (2016), approximately 0.28% of the population in Chhattisgarh is at high risk of suicide, with the highest risk group being 18–29-year-olds. ^15^ As per the NCRB, factors which contribute to suicide in Chhattisgarh include financial difficulties among men such as bankruptcy or indebtedness, and marriage related problems among both men and women. ^4^ The mental health treatment gap is also high in Chhattisgarh, ranging from 54.5 % (for psychosis) to 80.1% (for common mental disorders). ^19^

*Intervention district selection* ^16^

Rajnandgaon is home to an estimated 1.8 million people, with 82.27% residing in rural areas. The population includes 26% Scheduled Tribe (ST) and 10% Scheduled Caste (SC) communities and shows a 52% work participation rate. As a larger district, Rajnandgaon consists of 6.02% of the state’s population. It comprises 9 blocks with 1 district hospital, 1 medical college, 10 community health centres, and 47 primary health centres.

Balod district is home to approximately 0.8 million people, with 88% living in rural areas. 31% of the population belong to ST communities, and 8% to SC communities. The district’s work participation rate is 51%, and it is divided into 5 blocks and 704 villages. The healthcare infrastructure includes 1 district hospital, 5 community health centres, and 30 primary health centres.

Both districts lack follow-up care for attempted suicide cases in healthcare facilities, a gap this study aims to address by training health workers placed at multiple levels of the health system in these districts.

*Control district selection*

Balodabazar and Mungeli are two predominantly rural districts in Chhattisgarh. Balodabazar has a total population of approximately 1.31 million, with 87.25% residing in rural areas. The district’s population includes 23.37% Scheduled Castes (SC) and 12.8% Scheduled Tribes (ST). The district is administratively divided into six blocks and is served by one district hospital, six community health centres (CHCs), and 31 primary health centres (PHCs).

Mungeli has a smaller population of approximately 0.70 million, with an even higher rural proportion (90.67%). Its population comprises 27.76% SC and 10.37% ST. Mungeli consists of three blocks and is served by one district hospital, three CHCs, and 28 PHCs.

Like Balod and Rajnandgaon, Balodabazar and Mungeli are characterized by a predominantly rural, socially disadvantaged population and limited health infrastructure.

### Participants

We will recruit adults (≥18 years of age), who present to emergency public health facilities (district hospitals, primary or community health centers) in Balod and Rajnandgaon districts with attempted suicide or are already admitted for attempted suicide within one week of the study start date. We will exclude individuals who are unable to communicate clearly due to medical conditions or speech/hearing disabilities despite reasonable accommodations, those unable to comprehend one of the local languages used by CHOs to deliver the intervention, and individuals who do not reside within the catchment area of Balod and Rajnandgaon Health and Wellness Centers (where trained CHOs are placed).

### Study interventions

CASP was developed specifically for individuals at risk of suicide by SNEHA Suicide Prevention Center (Chennai, India) ^8^ and incorporates two evidence-based components: brief contact ^7, 11^ and safety planning. ^9^ Its core focus revolves around establishing periodic connections with individuals who have recently survived suicide attempts, alongside a collaborative effort to create and track progress using personalized safety planning cards intended for distressing as well as crisis situations. Within this framework, emergency nurses will report details (name of health facility, age and gender of participant) to the project team who communicate this to Community Health Officers (CHOs) from that locality. A nearby CHO will then follow up with and visit the consenting individual at a time and place of their choosing, within the upcoming week. The timing of these visits will adhere to the contact schedule outlined in the multi-site WHO suicide study, SUPRE-MISS (1, 2, 4, 7, and 11 weeks after the attempt). ^10, 12^ During these visits, CHOs will extend emotional support, gauge the level of risk, and jointly work with the individual to identify personal warning signs, coping strategies, and available support mechanisms (safety planning).

Safety planning ^8, 9^ will be carried out by the CHO with the participant in a quiet and confidential setting after each participant has received their own safety planning card. Safety planning will be a collaborative exercise wherein the CHO will guide the participant through each step involved in safety planning and help the participant to fill in the card. Participants will be encouraged to refer to their safety planning card when they are distressed, and their use will be monitored during visits. The primary objective of the safety planning card is to support participants when they experience suicidal thoughts and urges by reminding them of coping mechanisms that make them feel better, people they can reach out to and actions they can take to make their environment safer.

Referrals to social benefits: Many suicides occur spontaneously in periods of crisis where there is a breakdown in coping mechanisms for life stressors, such as financial difficulties, sudden illness or managing a chronic illness, relationship difficulties, etc. According to the National Crime Records Bureau (2022), family issues and illness are the main contributors to suicide in India, accounting for 32% and 18% of all suicides in 2022 respectively, while ‘Unemployment’ accounted for 1.9% and ‘Bankruptcy or indebtedness’ accounts for 4.1% suicides. ^4^ Thus, increasing access to state provided social benefits and schemes which focus on unemployment and livelihood support can help address some of these risk factors. CHOs will be trained to refer individuals in need of social security and support (e.g., government-provided employment or pension benefits, disability schemes, etc.) to the appropriate government workers, and all CHOs will have a list of locally available schemes and programs which they can use to match people to schemes and provide further information during visits.

### Control condition: Enhanced Usual Care (EUC)

In Chhattisgarh, usual care under the DMHP includes a telephonic follow-up, where a social worker or a clinical psychologist at the DMHP clinic provides counselling for an individual who has recently attempted suicide, and invites them to visit the DMHP clinic in person to receive continued support. However, some clinics (such as in as our intervention districts) do not have any existing follow up care for suicide due to staff shortages, and so the CASP pathway will serve as the primary follow-up response in these districts. In the control districts, participants will receive Enhanced Usual Care (EUC). EUC will consist of the usual care provided by the District Mental Health Plan (DMHP) in Balodabazar and Mungeli, which have an active DMHP program staffed by a social worker, a nurse as well as a psychiatric nurse, a clinical psychologist and a Medical Officer (MO) (a medical professional based in primary and community care clinics). DMHP personnel in control districts are trained in telephonic counselling for mental health and suicide which they apply while carrying out follow-up calls for suicide attempts. Moreover, the DMHP in the control districts carry out monthly mental health awareness raising camps in the community to increase awareness around mental health and their services (this is not occurring in the intervention districts). The CASP team will also provide a brief training with DMHP staff in control districts covering suicide, its prevalence and associated risk factors, and myth busting around suicide prevention.

### Recruitment & Retention

Individuals presenting at district hospitals, Primary Health Centres (PHCs), or Community Health Centres (CHCs) following a suicide attempt will be identified by casualty nurses, who will screen for eligibility and invite those meeting the criteria to participate in the study. After providing a brief explanation of the study, the nurses will obtain verbal consent from interested individuals and notify the research team via telephone, sharing details such as the participant’s village of residence and hospital discharge date.

The research team will then assign a Community Health Officer (CHO) from the nearest Health and Wellness Center to follow up with the participant at a time and place of their convenience. Within one week of discharge, the CHO will meet the participant, obtain written informed consent, and initiate follow-up care as per the CASP intervention protocol.

During the informed consent process, the CHO will explain the study’s purpose, procedures, and the potential risks and benefits of participation, emphasizing that involvement is entirely voluntary. Participants will receive a consent form and an information sheet detailing the study objectives, procedures, and the contact information of CASP research team members, enabling them to seek clarifications or address concerns at any point during the study.

To support participant retention in the study, we will make up to five contact attempts over a two-week period following each scheduled follow-up appointment. These attempts will include a combination of telephone calls and in-person home visits (where feasible and safe).

If a participant misses a follow-up but can be contacted later, they will still be approached for subsequent follow-ups at later time points.

For participants who actively request to discontinue follow-ups, the study team will first explore and address any concerns to encourage continued participation. However, if the participant continues to decline, they will be formally withdrawn from the intervention and no further contact will be made.

### Sample size

Since the present study is a pilot study (focused primarily on understanding feasibility and refining processes in anticipation of a full-scale trial), the sample size calculation is based on a conservative assumed rate of the secondary outcome (change in suicide attempts) of 10% at the end of 12 months in the control group. Thus, a minimum enrolment of 500 participants (250 control; 250 intervention) has a power of 80% (at a 2-sided alpha level of 0.05) to detect an absolute risk reduction of 10 percent points (minimal detectable difference of 10% between the groups (intervention and control), while allowing for an expected 20% loss to follow-up over the 12 months follow-up period.

### Training

All emergency nurses and Community Health Officers (CHOs) in the selected districts will be required to undergo CASP training, organized by the state government. The training sessions will be conducted by Master Trainers, a cadre of Rural Medical Assistants (RMAs) who will first receive a three-day training led by the study’s Principal Investigators (SP and LV). RMAs, trained under the Chhattisgarh RMA scheme, are formally qualified medical personnel working across Health and Wellness Centres in the state.

CHOs will participate in a two-day training program covering key topics such as understanding suicide and associated risk factors, empathetic communication, safety planning, suicide risk assessment, contact procedures, data recording, privacy and confidentiality, emergency response protocols, and self-care strategies to mitigate burnout.

Emergency nurses from all Primary Health Centres (PHCs), Community Health Centres (CHCs), and District Hospitals will undergo a one-day training delivered by Master Trainers. This training will focus on understanding suicide and its risk factors, empathetic communication, and obtaining verbal consent for study participation.

Additionally, a one-day refresher training will be provided to all health workers six months after the initial training to reinforce key concepts. Training materials will also be made available to health workers throughout the study to support their ongoing learning and implementation of CASP protocols.

### Supervision

In-person supervision will occur once a month in the districts and will be carried out by the research team. At supervision meetings, CHOs who are conducting follow-ups and nurses at the hospitals will be invited to meet with the research team and discuss challenges in implementation and carrying out of project tasks. Implementation of the project activities will be supported by the Master Trainers who are based in health facilities in the chosen districts, as well as by the District Coordinators who oversee CHOs in the districts.

### Outcomes

As this is a pilot study, the primary outcomes of interest are related to its implementation, specifically: reach, adoption, implementation (including cost), feasibility and acceptability, as summarized in Table 1. The RE-AIM framework will serve as the guiding conceptual framework for the primary outcomes in this study. ^20, 21^ Secondary outcomes include suicidal behaviour (including intention, ideation, planning and fatal or non-fatal suicide attempt) and symptoms of depression over a 6- and 12-month follow-up period.

**Table 1.** Implementation outcomes, indicators and timepoints of data collection.

Outcome	Indicator	Data source	Data collection
**Reach**	1) Absolute number and proportion of people who agreed to join the study	Study data (Nurses records)	Process data (during study period)
	2) Participant attrition/retention rate at endline (5 sessions)	Study data (CHOs records)	
	3) Demographic characteristics of participants compared to non-participants (Refusals and Lost to Follow up cases ^1^)	Study records	
**Adoption**	Nurses: Number and % of health centres where participants were identified and recruited	Study records	Process data (during study period)
	CHOs: Number and % of trained CHOs who deliver the CASP intervention		
**Implementation**	Of intervention: Proportion of CASP sessions which were delivered as per CASP intervention delivery schedule (on time)	CHO records	Process data (during study period)
	Of training: Change in knowledge, attitudes and skills of delivery agents (CHOs)	Pre-post survey of knowledge, skills and attitudes	Pre-pilot and at 18 months (sustained impact)
	Intervention costs, costs of (healthcare) resource use and quality of life	TiC-P and EQ-5D-5L with random sub-sample of 50 participants per condition (n=100)	Baseline and 6 months follow up
**Feasibility**	Extent to which health workers felt CASP is feasible to deliver	Semi-structured interviews with a purposive sample of health workers (n=20)	After concluding participant recruitment
**Acceptability**	Extent to which participants felt CASP is acceptable	In-depth interviews with a random sample of n=20 participants per district (total n=40)	After final contact point

^1^ Lost to Follow Up refers to individuals who have provided verbal consent to nurses in emergency wards, but with whom we have not been able to make subsequent contact and so therefore have not been formally recruited into the study.

*Primary outcome measures*

A brief survey collecting data on age, gender, education, marital status, employment status, caste/tribal background and income will be used to collect participant demographic information.

**Reach** will be measured by the absolute number, proportion, and representativeness of individuals who participate in CASP and how representative participants are compared to the target population. **Adoption** will be measured by the absolute number and proportion of health facilities and intervention agents who are willing to initiate and carry out CASP.

**Feasibility** will be measured using semi-structured interviews exploring the perceptions of health workers and supervisors on the extent to which CASP is viable as a suicide prevention response within the health system. Within these interviews, we will also explore the feasibility of health system adoption beyond the study, e.g. what supervision pathways can be established for maintenance and scale-up of the programme (as during the pilot, supervision will be carried out by the research team).

**Acceptability** of intervention delivery will be measured qualitatively via the same interviews with health workers and supervisors specifically to assess comfort with the CASP training and intervention content. Acceptability for participants will be measured qualitatively via in-depth interviews with a random sample of n=20 participants per district (total n=40) after their last session of CASP which will explore participant comfort with CASP session content and structure and to solicit feedback for adaptations to the intervention.

**Implementation** is defined as 1) the intervention agents’ adherence to the CASP schedule of session delivery (1, 2-, 4-, 7- and 11-weeks contact points), 2) the impact of the CASP training program and 3) the costs of the intervention. Timeliness of CASP sessions as delivered by CHOs will be measured via program data, specifically CHO records. Knowledge and skills improvement due to the CASP training will be measured by a pre-post knowledge and skills assessment with nurses and CHOs.

**Cost** will be assessed from the health system (costs required to run the program), patient (healthcare expenses, quality of life), and societal (e.g. productivity losses) perspectives with a sub-sample of participants from the main participant pool in both arms. The sub-sample will be selected using a simple random number generator, ensuring every participant in the main sample has an equal probability of inclusion and minimizing selection bias. If a participant has been selected as part of the cost sub-sample, the cost data will be collected along with the rest of the study data that is relevant at that timepoint.

The health system costs will be stratified based on the agencies incurring the cost and the nature of the cost (start-up capital cost and recurrent costs). The random sub-sample of participants in the intervention and control groups (n=100, 25 per district) will be asked about their **medical costs and productivity losses** in the previous four weeks using a modified Treatment Inventory of Costs in Patients (TIC-P) (Hindi) ^22^ at baseline and at 6 months follow up.

The TiC-P is a reliable instrument for collecting data on healthcare utilization and productivity loss in patients with mental health problems. ^22^ **Quality of life** will be assessed using the Hindi EuroQol-5D-5L (EQ-5D-5L) at baseline and at 6 months follow up. The EQ-5D-5L is a quality-of-life measure which has been widely used in India. ^23^ It measures quality of life via five dimensions, namely mobility, self-care, usual activities, pain/discomfort and anxiety/depression. Each dimension is measured by a Likert scale containing five levels spanning from a no problem to an extreme problem level.

*Secondary outcome measures*

1. Suicidal behavior

A modified Hindi version of the Columbia Suicide Severity Rating Scale (C-SSRS) Modified Screener Version ^24^ will measure suicidal behavior (suicidal intention, ideation, planning and fatal and non-fatal suicide attempt) at baseline and 6- and 12- month follow up. The Modified C- SSRS Screener is a semi-structured tool which measures the risk of suicide by inquiring about suicidal behaviour in the person’s lifetime (baseline) and since the last contact point (follow up). The screener consists of 6 questions and can be completed in a few minutes. For the purposes of this study, a lifetime measure was added to the screener to collect data on previous history of suicide ideation and behaviour at baseline. Specifically, the tool explores:

1. The presence of thoughts about suicide (suicidal ideation),
2. Actions taken by the individual, in relation to preparing for suicide,
3. Instances when the person has either attempted suicide or initiated a suicide attempt that was interrupted by another person or halted voluntarily, and the timing of this (lifetime, since last contact).

The C-SSRS has been translated in over 60 countries (including Hindi, and in India) and takes approximately 5 minutes to complete when administered as a clinical interview. The scale can be used by non-specialists across a range of settings, including healthcare settings.

2. Symptoms of depression

The Hindi Patient Health Questionnaire (PHQ-9) ^25^ assessment can be used as a screening tool as well as measures of symptom severity for depression. The nine-item PHQ-9 uses a 4- point Likert-scale with items ranging from 0 (not at all) to 3 (nearly every day). The PHQ-9 has been validated, widely used, and found to be suitable for use in India. ^26^

### Data collection

Baseline demographic data—including age, gender, education, marital status, employment status, caste/tribal background, income, and contact details—will be collected by Community Health Officers (CHOs) from consenting participants. Study records will include the following: (1) record books maintained by nurses at health facilities, documenting the date, age, gender, method of attempt, verbal consent status, and, if applicable, reasons for refusal; and (2) CHO records detailing the dates and locations of participant visits to monitor adherence to the CASP intervention schedule (at 1, 2, 4, 7, and 11 weeks post-attempt).

For outcome assessments, the Columbia-Suicide Severity Rating Scale (C-SSRS) and the Patient Health Questionnaire-9 (PHQ-9) will be administered to participants in the intervention group at baseline and during follow-ups at 6 and 12 months. Within the control arm, a brief follow up survey will be administered that captures suicide re-attempt at 6 and 12 month follow up points. Data collection will be conducted by trained data collectors, who are local Master’s graduates in mental health or related fields with prior experience in the health system.

To support the cost analysis and economic evaluation, a brief cost survey (Modified TiC-P) and the EuroQol-5D-5L will be administered to a random sample of 50 participants in both the intervention and control groups at baseline and at the 6-month follow-up. Data collection will be conducted using paper-and-pencil assessments or self-administered forms, based on participant preference.

**Figure 1.**
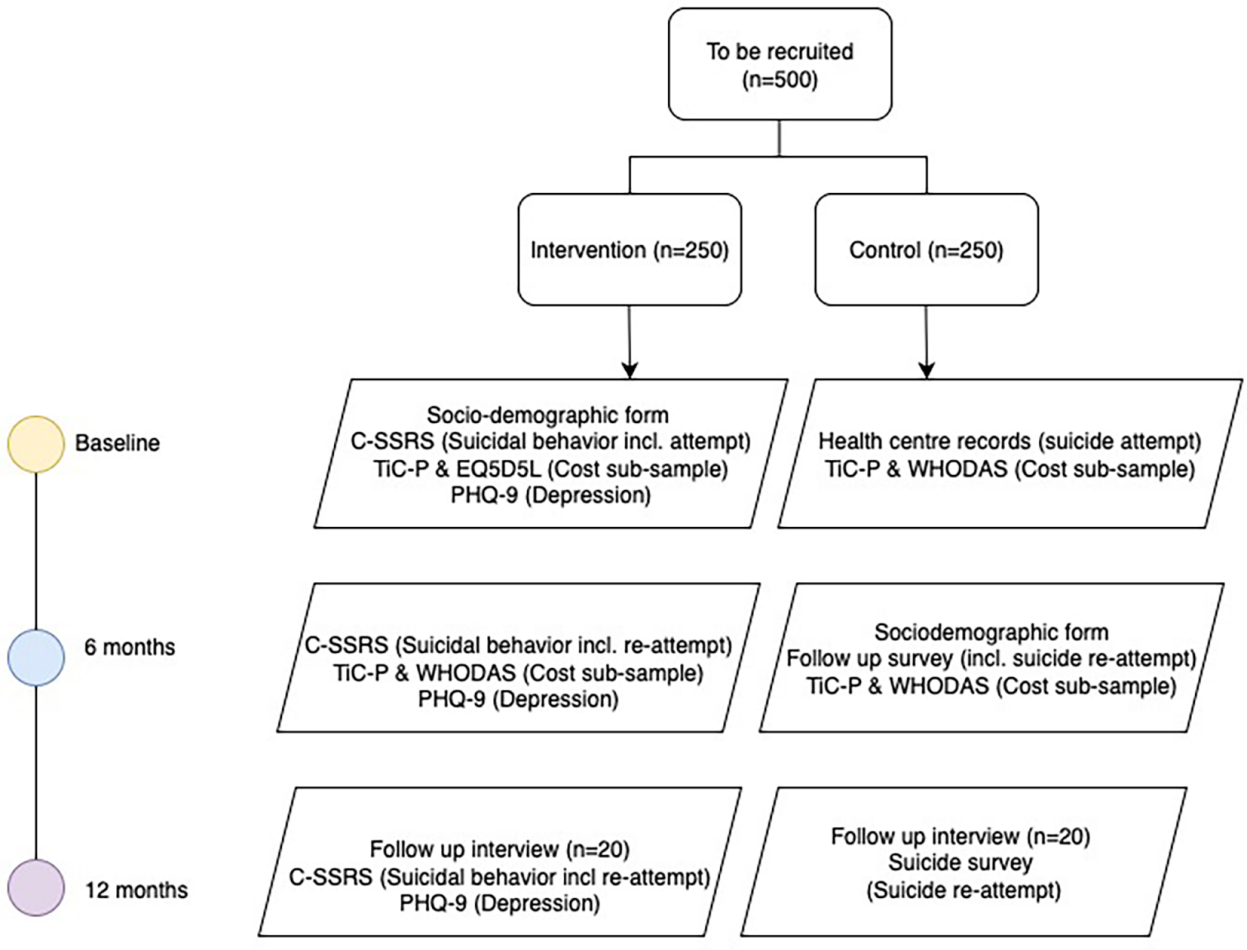
Flowchart of research procedures.

Qualitative follow-up data will be collected through in-depth and semi-structured interviews, lasting approximately one hour each. These interviews will be conducted by research team members and audio recorded with prior informed consent for transcription purposes. Acceptability interviews with participants will include a random sample selected independently of intervention completion status, ensuring the inclusion of individuals who may have dropped out of the study. See Figure 1 for research and data collection procedures.

### Data analysis

*Implementation outcomes*

Descriptive statistics (means, medians and standard deviations) of the reach, adoption and implementation metrics will be calculated. Qualitative thematic analysis ^27^ of the in-depth interviews exploring feasibility and acceptability will be carried out using a mix of inductive and deductive approaches. Two coders will independently review transcripts, generate initial codes, and collaboratively develop a comprehensive codebook (initial coding). This will be followed by a pilot coding phase to ensure procedural consistency before independent coding of the remaining transcripts, with discrepancies resolved through consensus or third-rater consultation. Researchers will then interpret and refine final themes. Coding will be done using NVivo 1.4.1.

*Clinical outcomes*

1. Difference in percentage of re-attempted suicide and death by suicide

We will analyze the difference between the intervention and control group in the percentage of participants who, after initial visit in the hospital for a suicide attempt, have re-attempted suicide (including deaths by suicide) at 6 and 12 months of follow-up. For statistical analysis, we will use Bayesian binominal testing as implemented in the open software program JASP. ^28^ Bayesian analysis allows us to not only test whether the null hypothesis is true or false, but also to test how likely the alternative hypothesis (that the change in suicide rates between intervention and control) is given the data. ^29, 30^

2. Change in depression, suicide risk and TiC-P scores

The mean change in depression and suicidal behavior scores between baseline and 6- and 12- months follow-up within the intervention group will be calculated using Bayesian linear mixed models as implemented in JASP. Models will be estimated both with and without a random effect for location, to see if it is needed to control for any clustering effect. ^31^

3. Quality of life

Analyses using EQ-5D-5L data will be presented in various ways. A basic subdivision will be made according to the structure of the EQ-5D-5L: 1) Presenting results from the EQ-5D-5L descriptive system as a health profile for each dimension; 2) Presenting results of the EQ VAS (visual analog scale) as a measure of overall self-rated health status, and 3) Presenting results from the EQ-5D-5L index values. The index values, presented in country specific value sets, are a major feature of the EQ-5D-5L instrument, facilitating the calculation of quality-adjusted life years (QALYs) that are used to inform economic evaluations of health care interventions. Mean change in QOL from baseline to end of follow-up will be presented separately in both control and intervention arms. An appropriate Bayesian linear mixed model using logit link and ordinal family will be employed depending on the data.

4. Economic evaluation

The cost analysis will evaluate the CASP program from health system (program implementation costs), patient (e.g., healthcare expenses, quality of life), and societal (e.g., productivity losses) perspectives. Healthcare expenses and productivity losses will be measured using the TiC-P, and quality of life will be assessed with the EQ-5D-5L. Implementation costs will be calculated by the research team based on start-up and recurring costs. Patient costs will be calculated using a bottom-up (micro-costing) approach: each item in the TiC-p will be multiplied by an appropriate unit cost and summed to provide an overall total cost. Given that references’ prices do not exists for India, unit prices will be determined based on previous studies ^32^ and international recommendations. ^33^ All cost prices will be converted to the 2024 price levels. Using EQ-5D-5L utilities, the area under the curve method will be used to compute quality-adjusted life years (QALYs) by using the utilities at baseline and 6 months follow-up (i.e. linear interpolation).

The economic evaluation will constitute a cost-utility analysis with QALYs as central outcome. To simultaneously evaluate both costs and outcomes, baseline-adjusted seemingly unrelated regression equations (SURE) models will be used. Given that costs are usually non-normally distributed the SURE models will be bootstrapped (2,500 times). To allow for intention to treat analysis, single imputation (based on predictive mean matching) nested in each bootstrap simulation will be used, as recommended by Brand and colleagues. ^34^ For each bootstrap simulation, incremental cost-effectiveness ratios (ICERs) will be calculated by dividing the incremental costs by the incremental QALYs, resulting in the costs per QALY gained. Next, ICERs will be plotted on a cost-effectiveness plane and a cost effectiveness acceptability curve (CEAC) will be constructed to determine the probabilities that the new intervention is cost-effective given a range of WTP values for a QALY. ^35^ Given the lack of formal WTP thresholds, a WTP ceiling of three times the national annual gross domestic product (GDP) per capita has been recommended. ^36^ Sensitivity analyses will be directed towards the main drivers of costs and QALYs.

### Data management and confidentiality

All participants will be identified by a Participant ID which is a code assigned to them when they enroll in the study. The only document which will contain both their name and their ID will be the Informed Consent Form. Signed informed consent forms will be kept in a locked cupboard in the Chief Medical Health Officer’s office in each district. After participants sign informed consent, all further forms will only contain their ID, not their name. A master sheet with all participant names and the corresponding IDs will be saved on a password-protected sheet which is only accessible by the research associates, project manager and PIs. Data entry will be done by research associates of the project.

### Potential harms

Any untoward medical occurrence, and/or (b) serious adverse events: suicide attempt, death due to suicide, death due to other causes, violence experienced by the participant, violence experienced by any other family member, and unplanned hospitalization will be reported. Although suicide attempt and death by suicides are outcome variables, we will also monitor them as if they are serious adverse events to ensure that the intervention is not associated with increase in suicide/attempted suicides.

### Protocol amendments

In the event of significant protocol modifications, our research team will promptly communicate changes in eligibility criteria, outcomes, analyses, and other vital aspects to the Indian Law Society Ethical Review Board in India, CTRI trial registry and Utrecht University, Netherlands Ethical Approval Board.

### Progression criteria

To determine if this study will proceed to a randomized controlled trial, we have developed progression criteria based on recommendations put forth by Mellor et al. (2023). ^37^ The progression criteria is focused on assessing the feasibility of the study design and the feasibility and appropriateness of the intervention, indicated in Table 2 below.

**Table 2.** Progression criteria for primary outcomes.

Outcome	Progression criteria
Feasibility of participant recruitment	Minimum recruitment rate of 50% of eligible participants
Feasibility of intervention design	• Retention of participants: <30% loss to follow up at 6-month data collection • Retention of CHOs: <20% of CHOs discontinue delivery of CASP sessions
Intervention implementation	Delivery of the intervention judged as highly feasible and acceptable by health workers and participants through qualitative data

These criteria have been selected considering practical limitations and contextual factors that may challenge the feasibility of integrating CASP into the existing health system, for e.g. lack of government resources to incentivize new tasks for health workers, significant distances to be travelled between facilities and participant homes (without adequate travel compensation) for CHOs, and stigma around suicide. As this is the first integration of CASP within the existing health system, one key focus of this study is to assess the impact of practical and contextual limitations and identify where and how we can adapt the study processes to better suit low resourced public health settings (where the scope for scale-up is largest).

Red Amber Green traffic light approach ^37^: Keeping the abovementioned contextual factors in mind, if after the completion of the pilot study the progression criteria are not met by more than 20% as per quantitative data, and the study is not found to be feasible as per qualitative data, this will be considered under the Red threshold and further exploration of this approach will not be considered. If the criteria are within a 20% range (lower than indicated), this will be considered within the Amber threshold and a reassessment will be carried out by the steering committee and relevant stakeholders, and appropriate adaptations of the intervention and trial processes will be made. If the criteria are met or exceeded by study data, then the intervention will be considered feasible and further assessment in a definitive trial may be explored.

### Dissemination

Dissemination of study findings will be done via the following channels: a) academic publications in peer reviewed, open access journals; b) development and dissemination of an easy-to-understand policy brief that highlights the study’s key findings and recommendations, geared towards policy-makers and stakeholders of the continued implementation or scale-up of CASP within the health system; and c) dissemination meetings within the districts in Chhattisgarh where the research was carried out, as well as among academic networks, healthcare professionals and relevant suicide prevention stakeholders.

### Study status

Participant recruitment and follow-up data collection for the CASP study is ongoing; data collection will conclude in November 2025.

## Discussion

This pilot study evaluates the implementation and preliminary effectiveness of a contact and safety planning (CASP) intervention delivered by trained health workers for suicide prevention in Chhattisgarh, India. While CASP has been previously tested among a refugee population in South India, ^8^ this study is the first to adapt and integrate CASP for an at-risk population within the public health system. By assessing the intervention’s feasibility, reach, acceptability, adoption, and early indications of effectiveness and cost, this study seeks to generate critical evidence for suicide prevention within the health system in low-resource settings.

Conducted in collaboration with the Chhattisgarh Department of Health and Family Welfare, CASP aims to address systemic challenges felt within the health system for suicide prevention in Chhattisgarh. For example, in the control districts of Mungeli and Balodabazar, while DMHP staff provide brief follow-ups and counseling for individuals who attempt suicide and present to emergency wards in district health centres, most other districts lack adequate personnel to fulfil these roles. As a result, attempted suicides often go unattended to. Additionally, nurses in emergency wards are not trained to identify and sensitively respond to attempted suicides, which can influence the care provided and medical decision-making in emergency settings. ^38^ Training emergency nurses and Community Health Officers (CHOs) as a key link between emergency departments—a common point of contact for individuals at risk—and the community provides a promising pathway to address these gaps.

We anticipate challenges when implementing this study. The unexpected doubling of the CHO trainee pool due to the placement of over 200 additional CHOs in district Health and Wellness Centres poses logistical difficulties for keeping to the study timeline and carrying out supervision and coordination with CHOs. Stigma around suicide within families and communities may hinder follow-ups and which are central to this model. Additionally, the lack of systematic data collection on suicide attempts and follow-ups in control districts complicates the evaluation. To mitigate this, data collectors will conduct weekly visits to engage with primary and community health centres and support in improving the consistency and quality of data collection and reporting at this level.

This study is timely and essential, given the growing global public health burden of suicide, particularly in low- and middle-income countries (LMICs). It addresses critical gaps in the literature regarding effective suicide prevention interventions in community settings and explores barriers and facilitators to implementation in under-resourced contexts. ^39^ Furthermore, it provides a practical perspective on the potential of health worker training at multiple levels to enhance suicide prevention efforts, and explores the ability for this programme to be scalable within and by the health system. Findings from this study will contribute to the definitive testing of CASP as a scalable intervention and inform strategies for integrating suicide prevention programs within public health systems in LMICs.

## Conclusion

The knowledge generated through this study will inform the future testing and scale-up of CASP across Chhattisgarh and other regions in India where specialist care and targeted suicide prevention initiatives are minimal. By examining factors influencing the successful implementation of CASP in public health systems, this study will support researchers, health professionals, and policymakers in addressing the suicide prevention gap in low-resource settings. It offers a crucial step toward reducing repeat suicide attempts and enhancing community-level responses to suicide in LMICs.

## Ethics and consent

Ethical approval has been obtained from Indian Law Society’s Institutional review board (ILS/173B/2022) dated 26 September 2022, the University of Utrecht Ethical Review Board, Netherlands (#23-0003) dated 1 October 2022 and the Clinical Trials Registry India (CTRI) (CTRI/2022/12/048087) for this study. All procedures will be done in adherence to guidelines outlined in the Declaration of Helsinki, including obtaining written informed consent from participants, ensuring confidentiality, and protecting participants’ rights, privacy and well-being. Written consent will be collected by Community Health Officers in the intervention districts and by data collectors in the control districts. Authorship of any publications which emerge from this project will be agreed upon by all authors based on individual contribution to the manuscript, project implementation, concept, steering, and evaluation.

## Author contributions

SK and SN drafted the manuscript. LV conceptualized the original intervention, SP and SK with guidance of LV designed the present intervention. SN, ST, LSZ, DdB, BW, LV and SP reviewed the manuscript, provided multiple rounds of edits and feedback, and approved the manuscript for submission. DP developed the plans for sampling and data analysis.

## Data Availability

Data collected as part of the study outlined in this protocol: Study data, after de-identification, can be shared with researchers whose proposed use of the data has been approved by an independent review committee. Data will be available 12 months after end of trial for up to 5 years and all requests for data must be directed to the Principal Investigators of the study. This protocol followed the SPIRIT guidelines. A completed SPIRIT checklist is attached in the Supplementary Materials along with this manuscript, and a SPIRIT figure can be found in the Zenodo depository. DOI: https://doi.org/10.5281/zenodo.14507570. ^40^ Data are available under the terms of the Creative Commons Attribution 4.0 International license (CC-BY 4.0).
