# Supplementary material for: Health worker delivered Contact and Safety Planning (CASP) for suicide prevention in Chhattisgarh, India: Protocol for a non-randomized, controlled pilot study
Source: F1000Res. 2025 Oct 6;14:279. Originally published 2025 Mar 10. [Version 2] doi: 10.12688/f1000research.159494.2 (PMC12508733; doi:10.12688/f1000research.159494.2)
Supplement: Peer review history

# Reviewer report: Reviewer response for version 2

**DOI:** 10.5256/f1000research.188309.r420844

**Published:** 2025-10-09

**Authors:** Nikki Lyka van Eijk

**Affiliations:** 1 Leiden University, Leiden, The Netherlands

I thank the authors for their detailed response and their adaptations to the protocol. I believe the article has improved significantly, with more clarity being provided on key points.

Is the study design appropriate for the research question?

Yes

Is the rationale for, and objectives of, the study clearly described?

Yes

Are sufficient details of the methods provided to allow replication by others?

Partly

Are the datasets clearly presented in a useable and accessible format?

Yes

Reviewer Expertise:

suicide, implementation

I confirm that I have read this submission and believe that I have an appropriate level of expertise to confirm that it is of an acceptable scientific standard.

# Reviewer report: Reviewer response for version 1

**DOI:** 10.5256/f1000research.175227.r397270

**Published:** 2025-08-20

**Authors:** Nikki Lyka van Eijk

**Affiliations:** 1 Leiden University, Leiden, The Netherlands

Dear editor,

 The authors describe a well-written study protocol for a brief intervention targeting suicide attempt survivors in Chhattisgarh, India. The choices made by the authors reflect that this is an initial pilot, with a potential randomized controlled trial following based on their results.

 The rationale of the study is clear, with high suicide rates in India overall, and the district being the fourth highest in the country. While this district has support in place in theory, the authors describe that logistical problems prevent the DMHP clinics from carrying out these services in several areas. An interventions such as CASP that does not require highly skilled individuals would allow for a more sustainable scale-up than the current system if proven effective.

 Considering that this is a pilot study, I believe the study design to be appropriate for answering the research questions. An ambitious combination of data sources is planned, including records, surveys, and interviews. The authors describe a sample size justification based on the secondary outcome. What is not entirely clear is how feasible this is to achieve within the time period. The authors have not provided data on how many people attend emergency departments in these districts following a suicide attempt. Adding this, as well as an estimate of what percentage of people would then enlist in the study, would help provide clarity on the feasibility of the study.

 In general, the methodology of the study is well-written and detailed, though mostly on the intervention group. As a reader, I have several questions regarding the control group that remain unanswered in this protocol. I would suggest the following changes:

- On p.4, a description is given of the two intervention districts. I would add a description of the control districts. Are they comparable in terms of population?
- Measures and data collection: it is not entirely clear which measures apply to both the intervention and the control condition. A flowchart of the research procedures could help clear this up. For example, if suicidal behaviour is measured using the CSSRS, and the authors state they wish to compare re-attempted suicide between groups, suicidal behaviour would have to be measured in the control group as well. At the moment, on p.8, the CSSRS is only mentioned for the intervention group.
- Implementation outcomes: the outcomes that are clearly defined as being assessed in both groups are the TiC-P and the EQ-5D-5L. Here, a random sample of participants in each group will be selected. I would recommend a clarification on the randomization process, as well as the recruitment of participants within the control group, since these participants will not have had the initial screening and informed consent process by a CHO.

On a sustainability and scale-up perspective, I have a concern about the supervision. Supervision is currently being carried out by the research team, which is understandable. However, if this approach shows promising results, districts will have to set up their own supervision structures after the research has ended. I recommend the authors already think about this at an early stage, to prevent a fade-out after the research team is no longer present to support implementation.

 I also have one minor remark: P.7 word is missing between measured and semi-structured.

 Overall, this study is relevant and timely. With some clarifications to the methods, I believe this protocol would be improved and a good starting point for future (hybrid) randomized trials.

Is the study design appropriate for the research question?

Yes

Is the rationale for, and objectives of, the study clearly described?

Yes

Are sufficient details of the methods provided to allow replication by others?

Partly

Are the datasets clearly presented in a useable and accessible format?

Yes

Reviewer Expertise:

suicide, implementation

I confirm that I have read this submission and believe that I have an appropriate level of expertise to confirm that it is of an acceptable scientific standard, however I have significant reservations, as outlined above.

# Response

**Published:** 2025-09-16

**Authors:** Sonali Kumar

**Affiliations:** CMHLP, Indian Law Society, Pune, Maharashtra, India

Dear Ms. van Eijk, 

 We'd like to sincerely thank you for your constructive feedback and conditional approval of our manuscript. We have carefully considered your suggestions and have revised the manuscript accordingly. Our detailed responses are: 

 “The authors describe a sample size justification based on the secondary outcome. What is not entirely clear is how feasible this is to achieve within the time period. The authors have not provided data on how many people attend emergency departments in these districts following a suicide attempt. Adding this, as well as an estimate of what percentage of people would then enlist in the study, would help provide clarity on the feasibility of the study.” 

- Response: Thank you for pointing this out. To clarify, when calculating the sample size for this study we considered population-level and facility-based data. Chhattisgarh has a suicide rate of 28.2 per 100,000 population, and suicide attempts are generally estimated to occur 20 times more frequently than suicide deaths. Given the combined population of Balod and Rajnandgaon districts is approximately 2.6 million, this corresponds to an estimated 14,600 suicide attempts annually. However, 2020–2021 state-level data from Chhattisgarh (unpublished) indicated that approximately 6,595 suicide attempts were recorded in public health facilities in Balod, suggesting that only a small proportion of attempts are presenting to or being recorded in health facilities. Using this data as a conservative proxy, we estimated that approximately 25–30% of attempts in the two study districts would present to public health facilities, resulting in 3,500–4,000 potentially eligible cases per year. Assuming 15–20% eligibility and consent, we anticipated that recruiting 500 participants over 12 months would be feasible. 

“On p.4, a description is given of the two intervention districts. I would add a description of the control districts. Are they comparable in terms of population?” 

- Response: Thank you; we have now included descriptions of the control districts in the Methods section (p.6).  

- Balodabazar has a population of approximately 1.31 million, with 87.25% residing in rural areas. The population includes 23.37% Scheduled Castes (SC) and 12.8% Scheduled Tribes (ST). The district is administratively divided into six blocks and is served by one district hospital, six community health centres (CHCs), and 31 primary health centres (PHCs).  

- Mungeli has a population of approximately 0.70 million, with 90.67% living in rural areas. Its population comprises 27.76% SC and 10.37% ST. Mungeli consists of three blocks and is served by one district hospital, three CHCs, and 28 PHCs. 

- Like Balod and Rajnandgaon, Balodabazar and Mungeli are predominantly rural, socially disadvantaged districts with limited health infrastructure, making them comparable to the intervention districts. 

“Measures and data collection: it is not entirely clear which measures apply to both the intervention and the control condition. A flowchart of the research procedures could help clear this up. For example, if suicidal behaviour is measured using the CSSRS, and the authors state they wish to compare re-attempted suicide between groups, suicidal behaviour would have to be measured in the control group as well. At the moment, on p.8, the CSSRS is only mentioned for the intervention group.” 

- Response: We understand and have clarified in the Aims section (p.5) and have added a table to the manuscript (p.13) that specifies which measures are applied to both intervention and control groups (and vice versa). We hope this helps with clarity of the study design.  

“Implementation outcomes: the outcomes that are clearly defined as being assessed in both groups are the TiC-P and the EQ-5D-5L. Here, a random sample of participants in each group will be selected. I would recommend a clarification on the randomization process, as well as the recruitment of participants within the control group, since these participants will not have had the initial screening and informed consent process by a CHO.” 

- Response: The cost sub-sample will be selected using a simple random number generator ( https://www.randomizer.org/), ensuring every participant in the main sample has an equal probability of inclusion and minimizing selection bias. 

- For recruitment in the control districts, data on hospital admissions for suicide attempts will be obtained from health facilities. Research assistants (RAs) will contact cases telephonically to introduce the project and obtain verbal consent. Data collectors will then follow up with patients at their home (or a preferred location, specified by them) to collect study data. If a participant has been selected as part of the cost sub-sample, the cost data will be collected along with the rest of the study data that is relevant at that timepoint.  

- We have now clarified both processes in the Methods section (p.11). 

“On a sustainability and scale-up perspective, I have a concern about the supervision. Supervision is currently being carried out by the research team, which is understandable. However, if this approach shows promising results, districts will have to set up their own supervision structures after the research has ended. I recommend the authors already think about this at an early stage, to prevent a fade-out after the research team is no longer present to support implementation.” 

- Response: We appreciate this important point and it is a key consideration for us as well. We have now included in the Methods and Discussion section (p.11, p.16) that a key objective of the pilot is to assess the feasibility of transitioning supervision from the research team to Master Trainers who are embedded within the health system. This transition will be explored through formative and follow-up interviews with participants and health system stakeholders to map a supervision pathway that is feasible for the programme’s continued adoption within the health system.  

We'd like to thank you again for your feedback on this paper and hope our responses have helped strengthen its clarity.

# Reviewer report: Reviewer response for version 1

**DOI:** 10.5256/f1000research.175227.r391622

**Published:** 2025-08-19

**Authors:** Seonaid Cleare

**Affiliations:** 1 University of Glasgow, Glasgow, Scotland, UK

This is an interesting protocol for a pilot study for a safety planning intervention.

 I like this study, and think the protocol is well written and detailed. I have a couple of minor pieces of feedback, which I hope will further strengthen the protocol.

1. Could the authors include a bit around participant retention strategies (e.g. how many attempts to contact the person for follow up will be made; if a person misses one follow-up, would they be contacted at the next time point?) please?
2. Additionally, it would be good to include details for withdrawal, loss to follow-up procedures. For instance, how many follow-up appointments would need to be missed before a person was recorded as lost to follow-up?
3. P9. Could the authors avoid using phrases such as “completed suicide” please?

I hope the authors find the feedback useful in revising their protocol.

Is the study design appropriate for the research question?

Yes

Is the rationale for, and objectives of, the study clearly described?

Yes

Are sufficient details of the methods provided to allow replication by others?

Partly

Are the datasets clearly presented in a useable and accessible format?

Not applicable

Reviewer Expertise:

emotional distress, self-harm and suicide

I confirm that I have read this submission and believe that I have an appropriate level of expertise to confirm that it is of an acceptable scientific standard.

# Response

**Published:** 2025-09-16

**Authors:** Sonali Kumar

**Affiliations:** CMHLP, Indian Law Society, Pune, Maharashtra, India

Dear Dr. Cleare,  

 Thank you for your thoughtful feedback on our protocol. Our responses to your comments are as follows: 

 “Could the authors include a bit around participant retention strategies (e.g. how many attempts to contact the person for follow up will be made; if a person misses one follow-up, would they be contacted at the next time point?) please?” 

- Response: Thank you Dr Cleare for this helpful suggestion. We have now included a description of our participant retention strategies in the Methods section (page 8). To clarify, to support participant retention in the study, we will make up to five contact attempts over a two-week period following each scheduled follow-up appointment. These attempts will include a combination of telephone calls and in-person home visits (where feasible and safe). If a participant misses a follow-up but can be contacted later, they will still be approached for subsequent follow-ups at later time points. 

“Additionally, it would be good to include details for withdrawal, loss to follow-up procedures. For instance, how many follow-up appointments would need to be missed before a person was recorded as lost to follow-up?” 

- Thank you for your question; we have now detailed our withdrawal (p.8) and loss-to-follow-up (p.10) procedures in the Methods section. For participants who actively request to discontinue follow-ups, the study team will first explore and address any concerns to encourage continued participation. However, if the participant continues to decline, they will be formally withdrawn from the intervention and no further contact will be made. Participants who cannot be reached despite five contact attempts over two consecutive follow-up periods will be recorded as lost to follow-up. 

“P9. Could the authors avoid using phrases such as ‘completed suicide’ please?” 

- Thank you for pointing this out; we have revised the manuscript to replace “completed suicide” with the preferred terminology “died by suicide” throughout the text. 

We hope these revisions address your concerns and strengthen the clarity of our protocol; once again thank you Dr Cleare for your constructive feedback.
